# Velarium control and visual steering in box jellyfish

**DOI:** 10.1007/s00359-013-0795-9

**Published:** 2013-02-16

**Authors:** Ronald Petie, Anders Garm, Dan-Eric Nilsson

**Affiliations:** 1 Department of Biology, Lund University, Biology Building B, Sölvegatan 35, 223 62 Lund, Sweden; 2 Marine Biological Section, Biological Institute, University of Copenhagen, Universitetsparken 4, 2100 Copenhagen Ø, Denmark

**Keywords:** Box jellyfish, Cubozoa, Velarium, Rhopalium, Vision

## Abstract

Directional swimming in the box jellyfish *Tripedalia cystophora* (cubozoa, cnidaria) is controlled by the shape of the velarium, which is a thin muscular sheet that forms the opening of the bell. It was unclear how different patterns of visual stimulation control directional swimming and that is the focus of this study. Jellyfish were tethered inside a small experimental tank, where the four vertical walls formed light panels. All four panels were lit at the start of an experiment. The shape of the opening in the velarium was recorded in response to switching off different combinations of panels. We found that under the experimental conditions the opening in the velarium assumed three distinct shapes during a swim contraction. The opening was (1) centred or it was off-centred and pocketed out either towards (2) a rhopalium or (3) a pedalium. The shape of the opening in the velarium followed the direction of the stimulus as long as the stimulus contained directional information. When the stimulus contained no directional information, the percentage of centred pulses increased and the shape of the off-centred pulses had a random orientation. Removing one rhopalium did not change the directional response of the animals, however, the number of centred pulses increased. When three rhopalia were removed, the percentage of centred pulses increased even further and the animals lost their ability to respond to directional information.

## Introduction

Box jellyfish are agile swimmers that use their elaborate visual system for orientation (Garm et al. 2007b). Steering in box jellyfish is accomplished by changes in the shape of the velarium (Gladfelter 1973). The velarium is a thin muscular sheet (Gladfelter 1973; Satterlie et al. 2005) that constricts the outflow opening of the bell during swim contractions. However, it is unclear how visual stimulation controls the shape of the velarium. In a previous study, we showed that the Caribbean box jellyfish, *Tripedalia cystophora*, responds to the darkening of a quadrant of the equatorial visual world by creating an off-centred opening in the velarium and delaying contraction in the quadrant of the animal next to the dark sector (Petie et al. 2011). In the present study, we investigate the shape of the velarium in response to different patterns of light and dark quadrants.

**Fig. 1:**
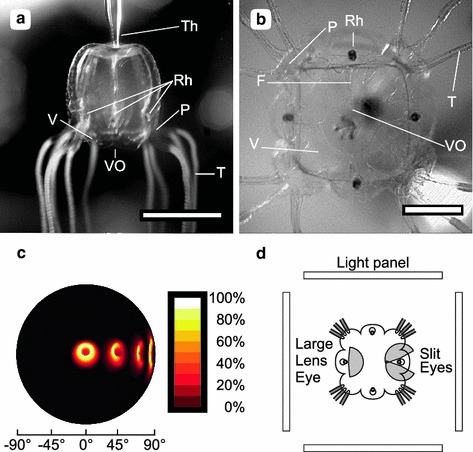
The box jellyfish *Tripedalia cystophora* in the experimental set-up seen from the side **a** and from below **b**, where **b** is an image from the high-speed sequence used for video analysis. **c** The field of view of the lower lens eye is illustrated by modelling the receptive fields of 4 individual photoreceptors. A central and 3 peripheral photoreceptors are shown. The rightmost receptive field corresponds to a photoreceptor on the edge of the retina and represents the outer edge of the field of view. The lower lens eye is rotationally symmetrical, which implies that the total width of the field of view is about 170°. The *colour map* shows the sensitivity of the receptors normalised to 100 %. The data used to make this figure can be found in (Nilsson et al. 2005). **d** A counter-intuitive feature of the visual system is the fact that the eyes viewing the visual scene below the animal are pointed towards the centre of the bell and obliquely downwards. Two eye types view the visual scene below the animal: the large lens eye and the paired slit eyes. For both eye types, the horizontal part of the field of view is indicated by *grey shading*. Note that the eyes point in the direction of the opposite bell wall and that each rhopalium views three light panels. Abbreviations: *F* frenulum, *P* pedalium, *Rh* rhopalium, *T* tentacle, *Th* tether, *V* velarium, *VO* velarial opening (*Scale bars* **a** 5 mm, **b** 2 mm)

Cubozoan jellyfish, or box jellyfish (Fig. 1), have a highly developed visual system (Claus 1878; Conant 1898; Berger 1900; Laska and Hündgen 1982; Yamasu and Yoshida 1976). They have 24 eyes clustered on four structures called rhopalia. Each of the clusters is identical in layout and has two lens eyes and four lens-less eyes. One of the lens eyes looks upward, while the other looks obliquely downward. The lens-less eyes come in pairs. One pair is slit shaped and aims obliquely down, while the other pair is pit shaped and points upward. The orientation of the rhopalium relative to gravity is kept constant because the rhopalium hangs on a flexible stalk and is weighed down by a heavy crystal at the bottom of the rhopalium (Garm et al. 2011). This means that the eyes that view the visual scene below the animal are the lower lens eye and the slit eyes, while the visual scene above the animal is viewed by the upper lens eye and the pit eyes. Note that the lower lens eyes and the slit eyes face inward and view the environment through the tissue of the bell (see Fig. 1d).

*Tripedalia cystophora* displays a couple of well-documented visually guided behaviours. The animals live in mangrove swamps in the Caribbean (Stewart 1996). Their habitat is penetrated by numerous prop roots, and light falling through the canopy creates shafts of light in the water. *T. cystophora* is attracted to these light shafts and feeds on the copepods that aggregate there (Buskey 2003; Stewart 1996). It also uses vision to avoid colliding with dark obstacles in the water (Garm et al. 2007b). By far the most complex visually guided behaviour in *T. cystophora* is navigation towards the edge of mangrove lagoons (Garm et al. 2011). This behaviour relies on the upper lens eyes of the jellyfish detecting the mangrove canopy through the water surface.

Box jellyfish use periodic contractions of the bell for propulsion (Shorten et al. 2005). Bell contraction generates a jet of water which propels the animal forward. In box jellyfish, the opening of the bell is constricted by a membranous, muscular sheet called the velarium (Gladfelter 1973). The velarium is suspended by four frenula, which are muscular triangular structures connecting the velarium to the inside of the bell (Gladfelter 1973; Satterlie et al. 2005). Some hydrozoan jellyfish have a similar structure to the velarium, called the velum, which has been demonstrated to increase swimming efficiency (Dabiri et al. 2006). In both cubozoan and hydrozoan jellyfish, the velarium, or velum, is involved in making the animal turn (Gladfelter 1973, 1972). In the current study, we investigated how different patterns of light and dark quadrants in the equatorial visual world affect the shape of the velarial opening and thus the direction of swimming. To approach this question, we tethered the animals in a small experimental tank, where the four vertical walls of the tank were fitted with light panels providing the visual stimuli.

## Materials and methods

### Animals

Animals were cultured at the University of Lund in Sweden and at the University of Copenhagen in Denmark. In total 33 animals were used. The size of the animals ranged from 0.43 to 0.89 cm. The mean bell diameter of the animals was 0.68 cm (SD 0.11).

### Experimental tank

During the experiments, the animals were placed in a set-up used in a previous study on steering in box jellyfish (Petie et al. 2011). Animals were tethered by the top of the bell, using a glass pipette with gentle suction, and placed in a Plexiglas tank with inside dimensions of 5 × 5 × 5 cm. The tank contained 25 ‰ sea water kept at 27 °C. The four vertical walls of the tank were covered with diffusing paper and a neutral density filter (transmittance 23.5 %). Each vertical wall was illuminated from outside of the tank by four blue-green LEDs (20410-UBGC/S400-A6, Everlight electronics co. ltd, Taipei, Taiwan). The diffuser was used to make a plane light source, while the neutral density filter was used to increase the contrast between lit and dark panels. Light emitted by a panel passed the neutral density filter once, while light reflected off the other panels had to pass the filter twice. Switching one or more panels off was used as the behavioural trigger. The colour of the LEDs matched the maximum spectral sensitivity of the animals and had a peak emission at 500 nm and spectral half width of 25 nm (Coates et al. 2006; Garm et al. 2007a). A box was placed over the set-up during the experiments to eliminate visual cues coming from outside, making sure that the eyes looking up through the water surface do not receive direct visual stimulation. Image sequences were recorded with a high-speed camera (MotionBlitz EoSens mini1, Model MC 1370, Mikrotron GmbH, Unterschleißheim, Germany) operated at 150 frames per second. Both the triggering of the camera and the light panels were controlled via a DAQ-card (NI USB-6229, National Instruments, Austin, Texas, USA) using a custom written program for LabVIEW 8.2 (National Instruments).

### Experimental procedures

For removal of rhopalia and for attachment of the suction pipette, animals were anaesthetized by immersion in a 1:1 mixture of sea water and magnesium chloride (0.37 M). Anaesthesia was performed outside the experimental tank, and care was taken to transport as little as possible of the magnesium chloride containing sea water to the experimental tank when transferring the animals. The animals were allowed to recover for at least 10 min before the experiments started. To test the effect of the anaesthesia, we counted the number of pulses for ten animals for a 3-min period before and after the application of anaesthesia. The average number of pulses before treatment was 161.7 (SD 53.7). When treating the animals for 3 min with anaesthesia, a 10-min recovery period was enough to restore the swim pulse count to 129.6 (SD 27.4). There was no significant difference in the number of pulses before and after the treatment (Paired *t*-test, *t* = 1.683, df = 9, *p* value = 0.13).

At the start of an experiment, the walls of the tank were lit for at least 5 min then one or more panels were switched off. We made sure that the combination of panels used as a stimulus was varied randomly. Light-off was chosen as a stimulus because this gave an immediate and clear response. No obvious responses were observed to increases in light intensity. The opening in the velarium did not get the off-centred shape associated with turning nor did we observe the large changes in swim pulse frequency that are seen after light-off.

Rhopalia were removed by cutting the stalk connecting the rhopalium to the bell. This is a minor procedure and we believe that removal of the rhopalium did not affect anything other than the visual-neural system of the animal. None of the muscles involved in swimming were cut and the bell was not damaged.

### Analysis

Image sequences were viewed using ImageJ version 1.47c (http://imagej.nih.gov/ij/). Analysis was done in R version 2.15.2 (R Development Core Team 2012) on RStudio version 0.96.331 (http://www.rstudio.org/) using the packages circular (Agostinelli and Lund 2011), CircStats (S-plus original by Ulric Lund and R port by Claudio Agostinelli 2009) and reshape2 (Wickham 2007). We measured 6 pulses for each animal. Sometimes, the animals pulsed less then 6 times in the recording time available on the high-speed camera. This is why the number of pulses used for the experiments varies. The shape of the opening in the bell of the animal was scored after visually assessing whether the out-pocketing was directed to one of the four rhopalia, one of the four pedalia or was centred. This method allowed us to determine out-pocketing direction with a 45° resolution. Sometimes, the shape of the velarial opening could not be resolved. These pulses were marked as “unresolved”.

**Fig. 2:**
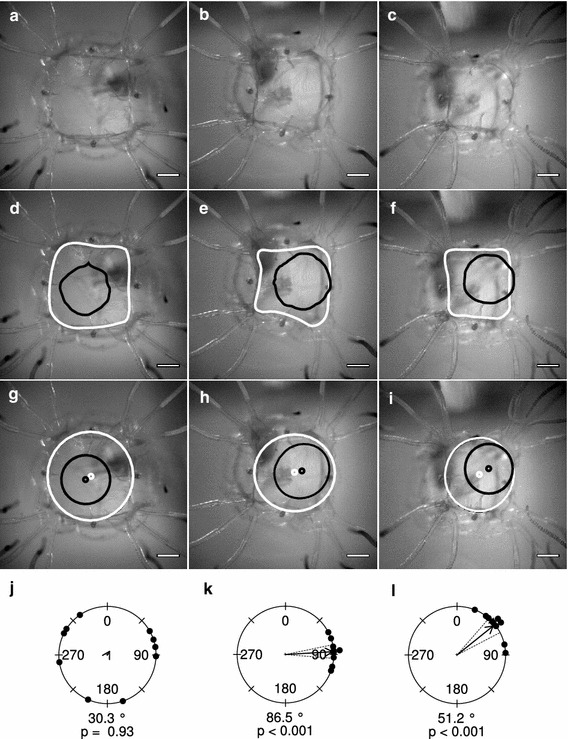
Shapes of velarial out-pocketing. During swim contractions, the opening in the bell of the velarium could assume three basic shapes. The opening in the velarium could be centred (**a**) or it could pocket out towards a rhopalium (**b**) or a pedalium (**c**). **d**–**f** To determine the direction of out-pocketing, we traced the inside of the bell (*white line*) and the opening in the velarium (*black line*). **g**–**i** Ellipses were fitted through the traces and the vector from the centre of the bell ellipse (*white*) to the centre of the velarial ellipse (*black*) described the direction of out-pocketing. From each of the three shapes, the direction was measured for one randomly chosen swim pulse for 10 animals. **j** Centred openings in the velarium had randomly directed swim pulses, while **k**–**l** off-centred swim pulses had a direction. Out-pocketing towards a rhopalium or a pedalium was significantly different from each other (Circular Analysis of Variance, df = 1, *F* = 23.14, *p* = 0.00014). The length of the arrow illustrates the length of the mean vector. The radius of the circle represents a length of 1

To determine the strength of our classification of the shape of the opening in the velarium into three categories, we randomly took 10 pulses from 10 animals for each category and measured the direction of the opening. We did this by tracing both the inside of the bell and the opening in the velarium (Fig. 2d, e, f). Following, we fitted ellipses through both traces (Fig. 2g, h, i). The vector from the centre of the “bell ellipse” to centre of the “velarium ellipse” was used to measure the direction of out-pocketing.

### Orientation

**Fig. 3:**
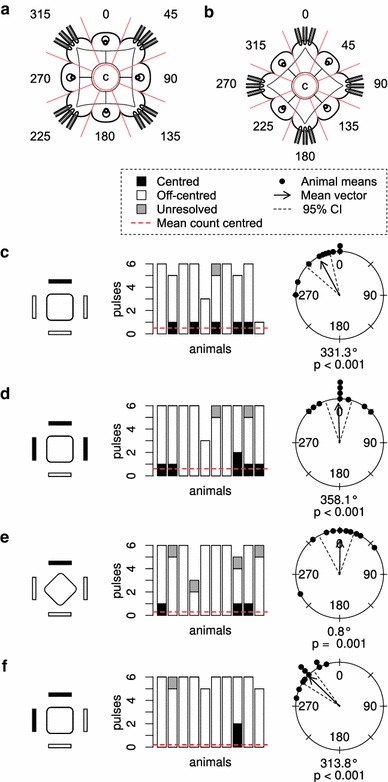
Directional stimulation. **a** and **b** show the two different alignments of the animal relative to the stimulus panels. The configuration shown in **a** is referred to as the “square” configuration and **b** as the “diamond” configuration. The animal is divided into eight 45° sectors for classification of the direction of off-centred out-pocketing. All experiments started with a period where all four stimulus panels were lit. The *black rectangles* in the drawings indicate the panels that have been switched off. The orientation of the *square* represents the orientation of the animal relative to the stimulus panels. The *stacked bar graphs* show the number of centred, off-centred and unresolved pulses for each animal. The *circle* *diagrams* show the out-pocketing directions of the off-centred pulses for each animal. The length of the arrow illustrates the length of the mean vector. The radius of the circle represents a length of 1. In **c** one panel was switched off and in **d** three panels were switched off. In **e** the animals were rotated 45° while one panel was switched off and **f** shows the experiment where two neighbouring panels were switched off. The direction of out-pocketing follows the direction of stimulation

In the experiments, we oriented the jellyfish in two different ways. In the “square” configuration (Fig. 3a), the four sides of the animal were parallel to the walls of the tank and the rhopalia faced the tank walls. Rotating the animals 45° resulted in the “diamond” configuration (Fig. 3b), where the sides of the animal made a 45° angle with the walls of the tank and instead the rhopalia faced the corner between two stimulation panels.

### Optical model

The receptive field of the lower lens eye was obtained by modelling. Rays were traced through an geometrical model containing refractive indices of the eye and the sizes and orientations of the photoreceptors. The optical model is described in detail in Nilsson et al. (2005).

## Results

For understanding the experiments, it is important to realise that when orienting the animal vertically as we did (see Fig. 1a), the lower lens eyes were pointed in the direction of the centre of the bell, while each eye of the paired slit eyes was pointed approximately in the direction of the corner between two panels (see Fig. 1b, d). Since, the animal is transparent it will in fact look through its own bell. The rhopalia faced the opposite panel, not the closest panel, and received input from the opposite panel and the two panels next to it. With the rhopalia in this orientation, the large lens eyes with their 170° field of view (Fig. 1c) looked directly at the opposing panels, while also viewing the panels to the left and right. Each slit eye has a 170° visual field (Garm et al. 2008) and viewed the opposing panel with approximately half the visual fields, while viewing the panel to the side with the other half of their visual field (see Fig. 1d).

### Types of velarial out-pocketing

We found that under our experimental conditions the opening in the velarium could assume three different shapes: (1) the opening in the velarium could be centred, (2) it could pocket out towards a rhopalium or (3) towards a pedalium (Fig. 2). For the pulses that we classified as directed towards a rhopalium, the mean vector had an average direction of 86.5° and a length of 0.97 (Fig. 2l). The expected out-pocketing direction of 90° is included in the 95 % confidence interval. Pulses classified as directed towards a pedalium had a mean vector with a direction of 51.2° and a length of 0.95 (Fig. 2k). In this case, the expected direction is 45° and is again included in the confidence interval. The mean out-pocketing directions of pulses towards a rhopalium or a pedalium were significantly different from each other (Circular Analysis of Variance, df = 1, *F* = 23.14, *p* = 0.00014). As expected, the pulses with a centred opening in the velarium had no apparent directionality with a mean vector having a direction of 30.3° and, most importantly, a length of only 0.086 (Fig. 2j).

### Directional stimulation

As shown in our previous study (Petie et al. 2011), switching off one panel produced a response where the pulses pocket out in the direction of the dark panel (Fig. 3c). The mean vector for this response had a direction of 331° (expected direction 0°) and a length of 0.88. Ten percent of the pulses was centred. For more details see Table 1. A comparable response is seen when three panels were switched off (Fig. 3d). In this case, the mean direction was 358° (expected direction 0°) and the length 0.89. Eleven percent of the pulses was centred. In the previous figures, the mean direction of the pulses was approximately directed towards a rhopalium. In Fig. 3e, f, the velarium pockets out towards a pedalium instead. For Fig. 3e, the mean direction was 0.8° (expected direction 0°, length 0.77, 5.4 % centred) and for Fig. 3f, this was 314° (expected direction 315°, length 0.95, 3.4 % centred). The response shown in Fig. 3f was very consistent. From our previous studies (Petie et al. 2011, 2013), we expected the opening of the velarium to be directed towards the centre of the dark panel(s). Only in the experiment where one panel was switched off (Fig. 3c), the expected direction was not included in the 95 % confidence interval.

**Table 1:** Pulse counts and circular statistics for the off-centred swim pulses

Type	Animals	Pulse counts					Mean vector		Rayleigh test	95 % confidence interval		
		Total	Centred	Off-centred	Unknown	% centred	Direction	Length	*p* value	CI1	CI 2	Figure
Square, 1 panel off	10	50	5	44	1	10	331.3	0.88	<0.001	311.4	345.7	3c
Square, 3 panels off	10	57	6	49	2	11	358.1	0.89	<0.001	341.0	14.5	3d
Diamond, 1 panel off	10	56	3	49	4	5	0.8	0.77	0.001	328.6	20.1	3e
Square, 2 neighbouring panels off	10	58	2	55	1	3	313.8	0.95	<0.001	303.1	324.0	3f
Square, all panels off	4	60	34	24	2	57	145.0	0.18	0.92	2.8	254.3	4a
_Diamond_, _2__opposite_ _panels_ _off_	9	51	30	21	0	59	15.5	0.18	0.78	264.2	126.9	4b
Square, 2 opposite panels off	10	57	13	42	2	23	351.8	0.31	0.44	170.4	78.3	4c
Square, constant light	10	43	8	35	0	19	276.6	0.60	0.023	243.5	321.3	4d
3 rhopalia, panel A off	9	53	7	38	8	13	351.2	0.91	<0.001	337.2	7.8	5a
3 rhopalia, panel B off	9	52	25	24	3	48	74.4	0.76	0.003	48.8	102.7	5b
3 rhopalia, panel C off	9	51	13	31	7	25	191.2	0.94	<0.001	178.3	204.7	5c
3 rhopalia, constant light	9	40	16	23	1	40	151.9	0.10	0.925	29.7	361.5	5d
1 rhopalium, panel A off	10	60	18	41	1	30	352.1	0.94	<0.001	340.3	5.6	6a
1 rhopalium, panel D off	10	56	25	31	0	45	338.3	0.70	0.004	306.0	11.1	6b
1 rhopalium, panel C off	_10_	_59_	_50_	_9_	_0_	_85_	_19.9_	_0.83_	_0.009_	_0.0_	_53.9_	6_c_
1 rhopalium, constant light	10	50	41	9	0	82	227.5	0.15	0.903	80.1	343.7	6d

The “Square” and “Diamond” configuration refers the orientations shown in Figs. 3a, b. For each experiment, the mean vector for the off-centred pulses was calculated. The Rayleigh tests if responses had random directions

### Undirectional stimulation

**Fig. 4:**
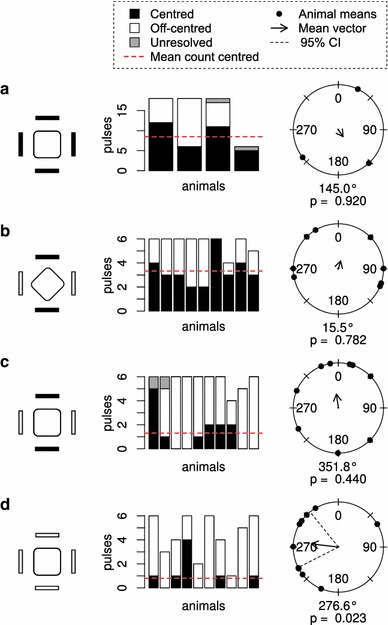
Undirectional stimulation. **a**–**c** Switching four panels or two opposing panels off resulted in responses with a higher percentage of centred pulses compared to the responses to directional stimulation. The off-centred pulses had a random direction. **d** At constant light a preferred out-pocketing direction remained. The figure reads as Fig. 3

Switching off all panels resulted in contractions where 57 % of the pulses were centred. (Fig. 4a; Table 1). The off-centred pulses did not have a significant direction (Rayleigh test, *p* value = 0.92). With the animal in the diamond orientation, switching off opposing panels very consistently produced contractions with a centred velarium opening. 59 % of the pulses were centred and the off-centred pulses had no direction (Fig. 4b, Rayleigh test, *p* value = 0.78). With the animal in the square orientation, 23 % of the pulses were centred, and again the off-centred pulses did not have a significant direction (Fig. 4c, Rayleigh test, *p* value = 0.44). Under continuous light, the animals produced bell contractions with a centred velarial opening in 19 % of the pulses (Fig. 4d). Despite the lack of directional stimulation, the pulses had a significant direction (Rayleigh test, *p* value = 0.023).

### Jellyfish with three rhopalia

**Fig. 5:**
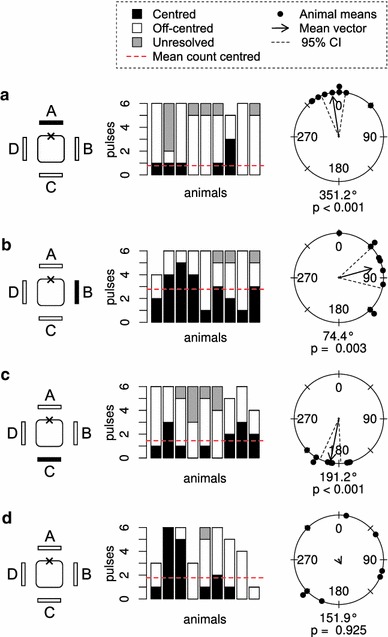
Animals with three rhopalia. **a**–**c** When a light panel at different relative positions to the removed rhopalium was switched off, the direction of the centred pulses still followed the direction of stimulation. The removed rhopalium is marked by an ‘*x*’. **d** At constant light the off-centred pulses lost directionality. The figure reads as Fig. 3

In animals with three rhopalia, the direction of the opening in the bell still followed the direction of stimulation. When panel A was switched off (Fig. 5a, expected direction 0°) the direction of out-pocketing was 351°, when panel B was switched off the mean direction was 74° (Fig. 5b, expected direction 90°) and when panel C was switched off the direction of out-pocketing was 191° (Fig. 5c, expected direction 180°). In all cases, the expected direction was included in the confidence interval (see Fig. 5; Table 1). Notice that the percentage of centred pulses increased when panel B was switched off. Under constant illumination, we saw an increase of centred pulses in some animals while the mean direction of the off-centred pulses was random (Fig. 5d, Rayleigh test, *p* value = 0.925).

### Jellyfish with one rhopalium

**Fig. 6:**
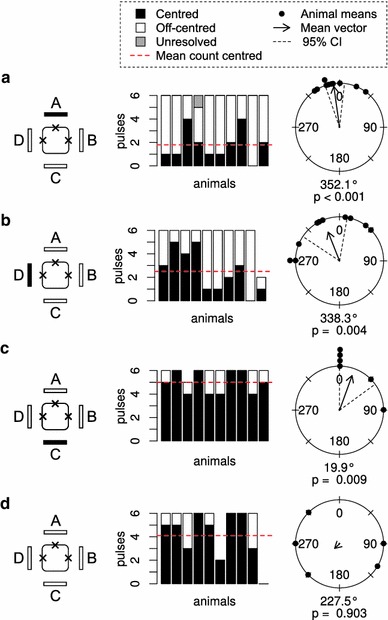
Animals with one rhopalium. **a** When the panel opposing the remaining rhopalium is switched off, the direction of the off-centred pulses is the same as for intact animals and animals with three rhopalia (Circular Analysis of Variance, df = 2, *F* = 1.945, *p* = 0.1632). The removed rhopalia are marked by ‘*x*’. **b**, **c** When switching off panel D or C the pulses are still directed toward panel A and the percentage of centred pulses increases. **d** At constant light the off-centred pulses lost directionality. The figure reads as Fig. 3

In all experiments on animals with one rhopalium, we saw an increase in the percentage of centred pulses. Interestingly, the response to switching off panel A is still remarkably like the response of an intact animal. In fact, no significant difference existed in mean out-pocketing directions between animals with one rhopallium, intact animals and animals with three rhopalia (Circular Analysis of Variance, df = 2, *F* = 1.945, *p* = 0.1632). Even when panel D or C was switched off, we saw that the opening of the velarium was still directed roughly towards panel A (Fig. 6b, c). When panel C was switched off, we saw the highest percentage of centred pulses recorded in these experiments of 85 %. Under constant illumination, animals also had a high percentage of centred pulses (82 %) and the direction of the off-centred pulses was random (Fig. 6d, Rayleigh test, *p* value = 0.903).

## Discussion

This study shows that the box jellyfish *T. cystophora* responds differently to different patterns of visual stimulation. We saw that the shape of the opening in the velarium followed the direction of stimulation, as long as the stimulus contained directional information. When the stimulus contained no directional information, the percentage of centred pulses increased and the shape of the opening in the velarium had a random orientation. Animals with three rhopalia retained the response to directional stimulation, however, the number of centred pulses increased. For animals with only one rhopalium left, the percentage of centred pulses increased even further and the animal lost its ability to respond to directional information.

### Eyes involved

The upper lens eyes and the pit eyes are most probably not involved, since the box covering the experimental tank removes visual cues from above; which is the direction from which these eyes collect their light (Garm et al. 2011, 2008). Only the eyes viewing the visual scene below the animal, the lower lens eyes and the slit eyes, had a direct view of the stimulus panels (Garm et al. 2008; Nilsson et al. 2005). Interestingly, these eyes are also believed to be involved in obstacle avoidance behaviour in this species of box jellyfish (Garm et al. 2007b), and it is likely that the behavioural responses investigated here are in fact the obstacle avoidance behaviour.

### Control of the shape of the velarium

**Fig. 7:**
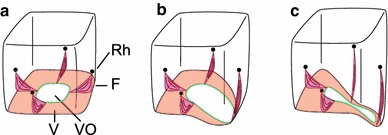
Proposed patterns of relaxation and stiffening of the frenula and velarium. Different shapes of the velarial opening are caused by different patterns of relaxation and stiffening of the velarium and frenula. For each of the three basic shapes of the velarium, an activity pattern is proposed. **a** A centred opening is most likely caused by contraction of the entire velarium. **b** Out-pocketing towards a rhopalium probably results from one frenulum relaxing, including a local relaxation of the velarium at that location. **c** Out-pocketing towards a pedalium is likely caused by relaxation of two frenula and relaxation of the corresponding half of the velarium

It seems logical that the pattern of activation of the muscles in the velarium determines the shape of the opening in the velarium. Contraction of all muscles in the velarium very likely gives the velarium a centred opening, as illustrated in Fig. 7a. Relaxing the muscles in one quadrant of the velarium is probably how out-pocketing towards a rhopalium is accomplished (Fig. 7b). Out-pocketing towards a pedalium looks less straight forward, but is likely the result of relaxation of half of the velarium. Stiffening and relaxation of the frenula probably coincide with the velarium, since muscles in the frenula are continuous with the velarium (Satterlie et al. 2005). The alternative mechanism to the relaxation mechanism is of course presented by enhanced contractions of the muscles in the areas of the velarium that are not pocketing out. Other muscles that could deform the bell, and thus change the shape of the opening in the bell are the radial bands of smooth muscle originating just above the rhopalia. However, involvement of these muscles seems unlikely. Smooth muscles typically are slow, and medusa can change the shape of the opening from centred to off-centred between pulses (Petie et al. 2011). It could be that each rhopalium directly controls the muscular activity of the closest quadrant of the velarium. On the other hand, involvement of the ring nerve seems likely since the ring nerve innervates the velarium (Satterlie 2011) and connects the rhopalia (Garm et al. 2007c). Involvement of the ring nerve is also suggested by experiments on animals with one rhopalium.

### Setting swim direction

From our previous study (Petie et al. 2011), we know that in response to darkening of one quadrant of the visual field the animals have swim pulses with an out-pocketing towards the darkened panel. This result is confirmed in Fig. 3c. It appears that when rotating the animal 45°, the opening of the velarium still pockets out towards the dark panel, illustrated in Fig. 3e. This shows that the animal can set its turning direction with an accuracy of at least 45°. The difference in stimulation caused by rotating the animal is resolved by the visual system. This task could be solved by a single eye. The 45° change in the angle of the stimulus direction falls well within the 10–20° spatial resolution of the large lens eyes (Fig. 1c) (Nilsson et al. 2005). However, as we will discuss later in more detail, animals with one rhopalium can no longer adapt the direction of out-pocketing to the direction of the stimulus. This argues in favour of the hypothesis that the rhopalia together set the direction of out-pocketing.

After bell contraction, the velarium pocketed inwards, and out of focus of the camera. In between swim pulses, the velarium had a width of about 10 % of the diameter of the bell.

Animals adjust the direction of the off-centred pulses readily to the direction of stimulation. When three panels (Fig. 3d) or two neighbouring panels (Fig. 3f) are switched off, the pulses still get directed to the centre of the dark panels.

When the animals were presented with undirected visual stimuli (Fig. 4), the number of centred pulses increased and the off-centred pulses lost directionality. Apparently when no directional information is present in the stimulus, the animals more often swim straight by having a centred opening in the velarium or turn in a random direction. It was unexpected to find that the off-centered swim pulses under constant light were not randomly oriented but had a direction (Fig. 4d). It could have been that the tethering procedure introduced an asymmetry in the bell in this experiment series. To minimize the effect of bias in the swim system, the combination of panels used for stimulation was randomly chosen.

Removing one of the four rhopalia gave us insight into the flexibility of the mechanism controlling the shape of the velarium. Animals were still found to be able to adequately respond to the direction of the stimulus, however, a slight increase in the percentage of centred pulses was observed. The loss of one rhopalium is almost completely compensated for by the other rhopalia.

Animals with only one rhopalium were able to respond almost as good as intact animals when the light panel opposite to the remaining rhopalium was switched off, as shown in Fig. 6a. When panel D or C was switched off, animals still responded by having pulses with the opening roughly directed towards panel A. In all experiments, there also is a clear increase in the number of centred pulses. Combining these two findings we pose the following ideas: (1) the “standard” shape of the velarium during a swim pulse is centred. (2) Visual input to the rhopalia controls the part of the velarium at the opposite side of the bell, likely via the ring nerve. Rhopalia communicate via the ring nerve to (3) increase the robustness of the system and (4) prevent unfavourable velarium shapes. An unfavourable shape would, for example, be when two opposing sides of the velarium relax. This would not contribute to steering and it would decrease swimming efficiency by increasing the size of the opening in the bell.

### Concluding remarks

In all of the experiments on intact animals, the animals responded in a way that would steer the animal away from the darkened panel(s). Assuming that the same mechanisms are at play in freely swimming animals, this would provide the animals with the means to avoid colliding with dark objects appearing in their field of view, and this corresponds neatly with the object avoidance response described previously (Garm et al. 2007b).

Finally, our behavioural data supports the idea that rhopalia control the velarium on the opposite side of the bell. Physiological experiments need to be done in the future to test this hypothesis.
